# Supplementary material for: Within-Season Changes in Land-Use Impact Pest Abundance in Smallholder African Cassava Production Systems
Source: Insects. 2021 Mar 22;12(3):269. doi: 10.3390/insects12030269 (PMC8005198; doi:10.3390/insects12030269)

Supplementary Information

Figure S1. Dendrogram showing the seven clusters in the landscape

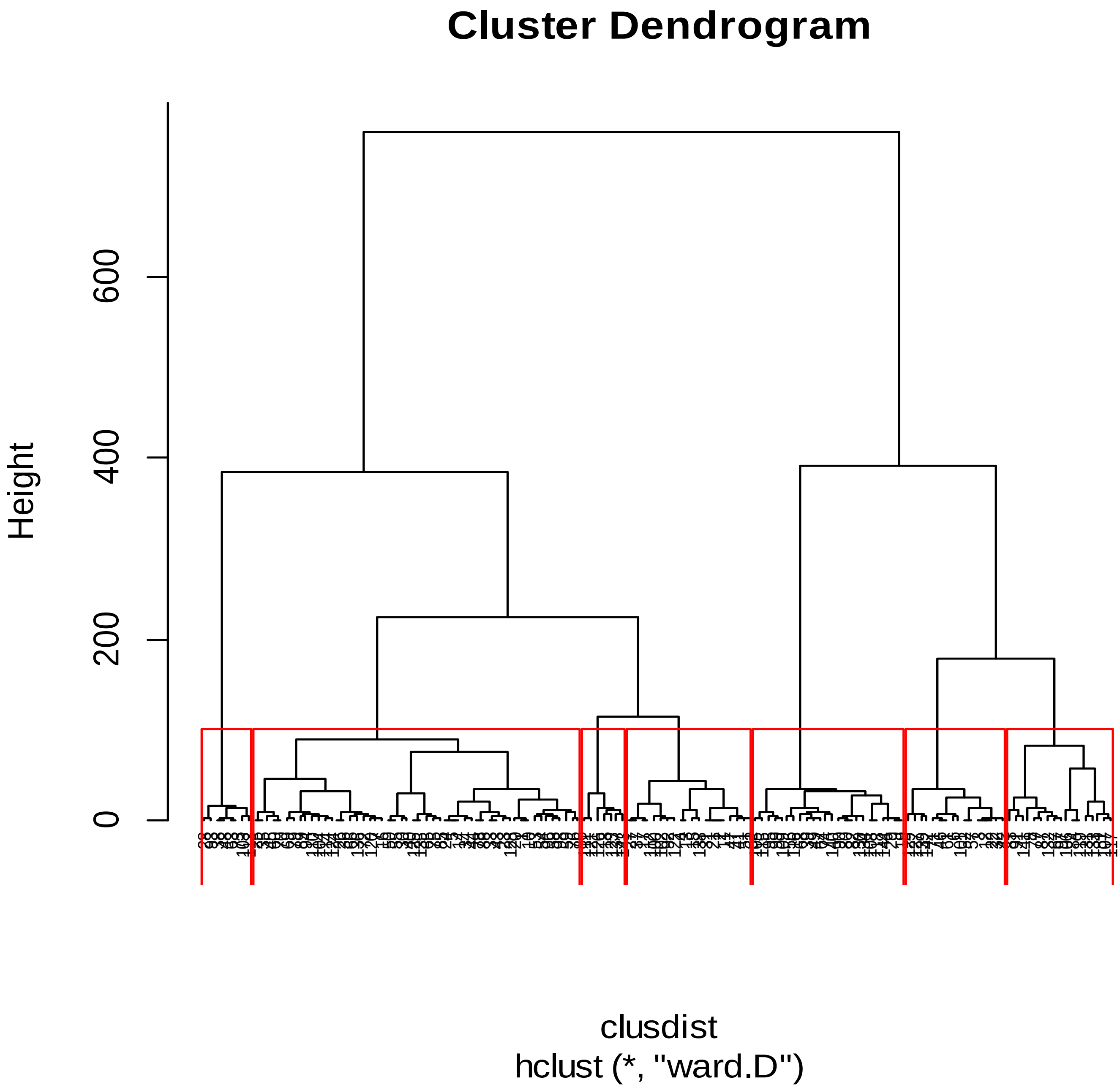

Figure S2. Correlation between SSA1 *Bemisia* nymphs and cassava age

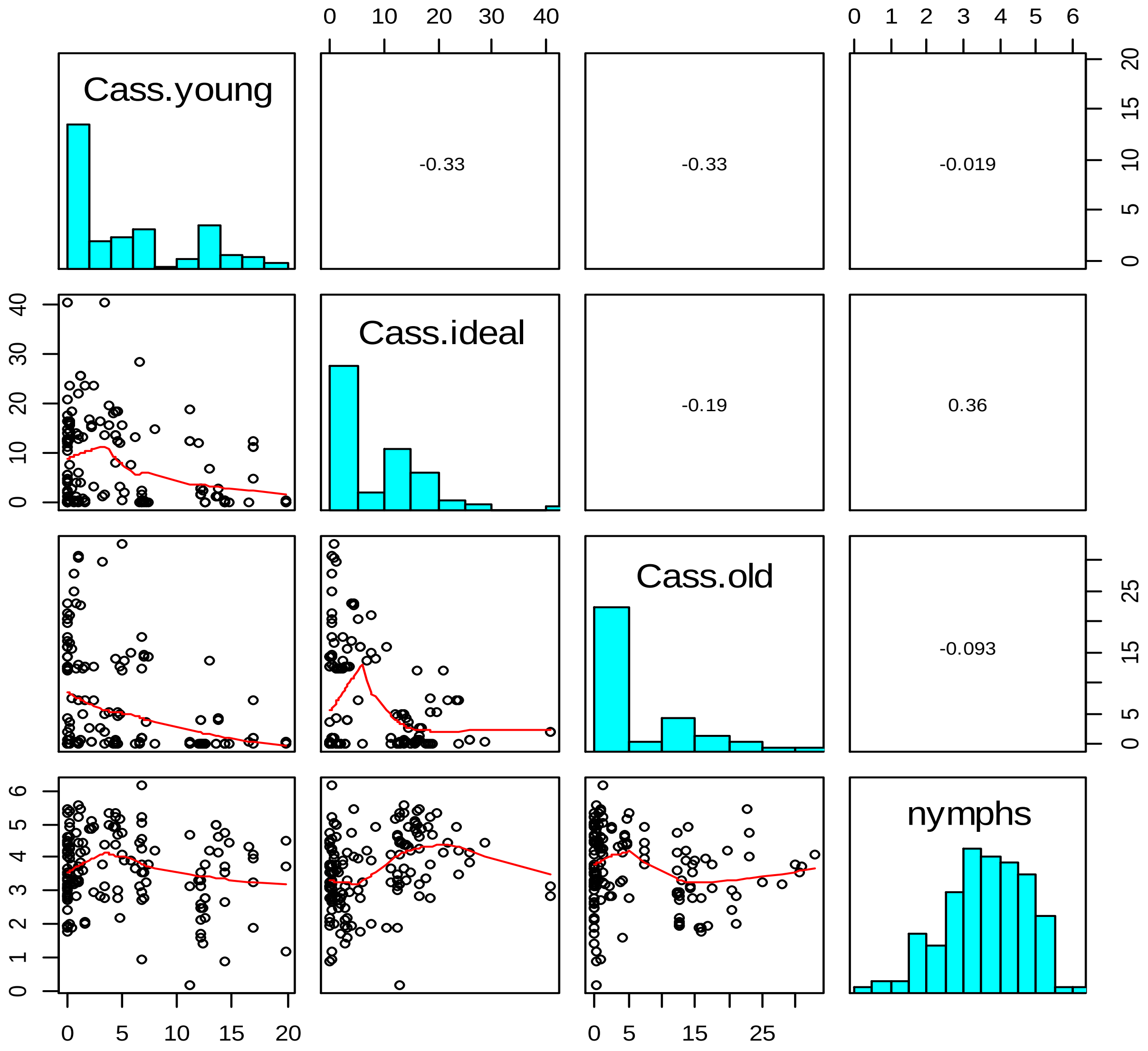

Figure S3. Correlation of SSA1 *Bemisia* nymphs with area of different crops in the landscape

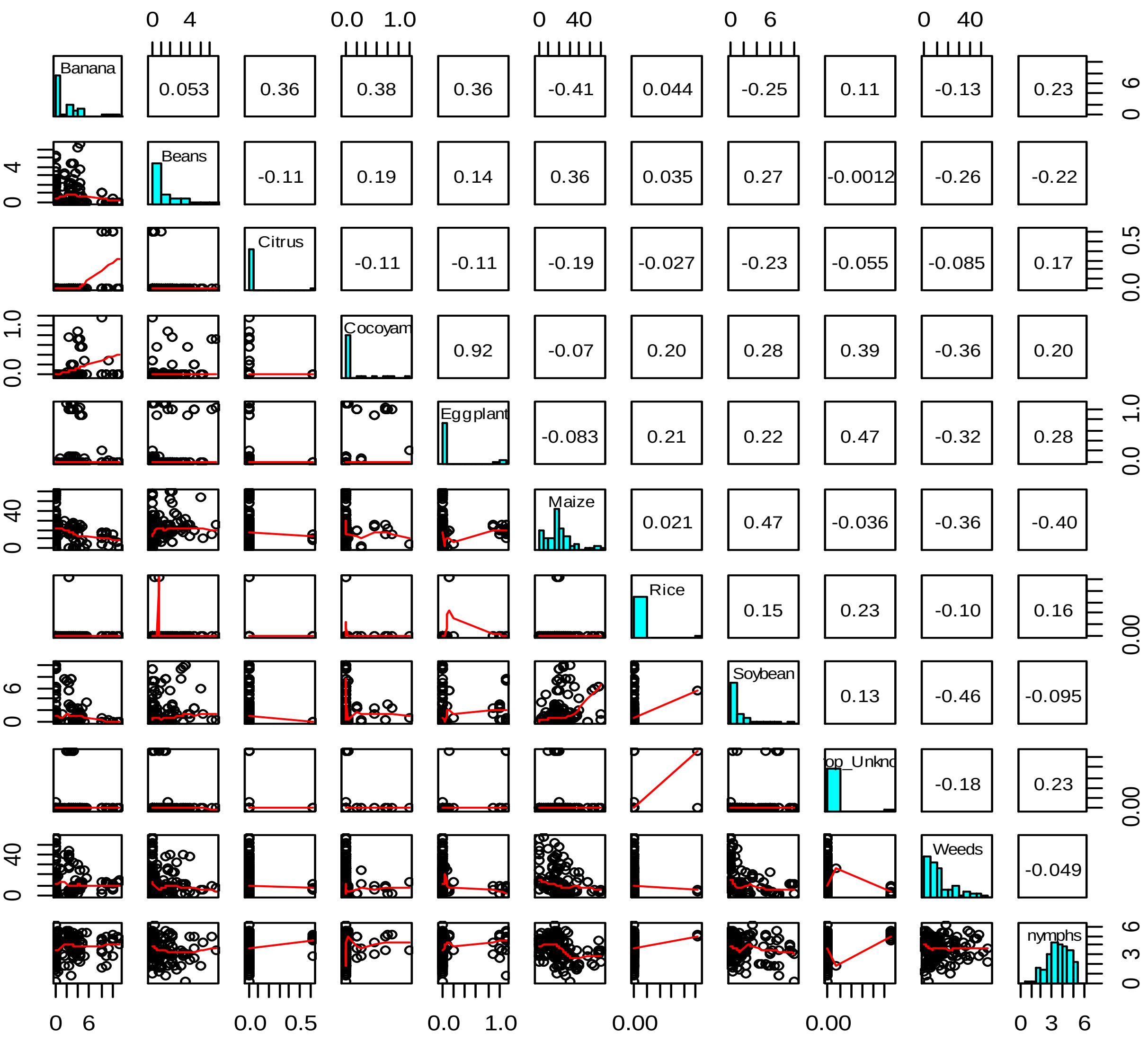

Figure 4 Relationship between area of soybean and *Bemisia* nymph abundance in cassava.

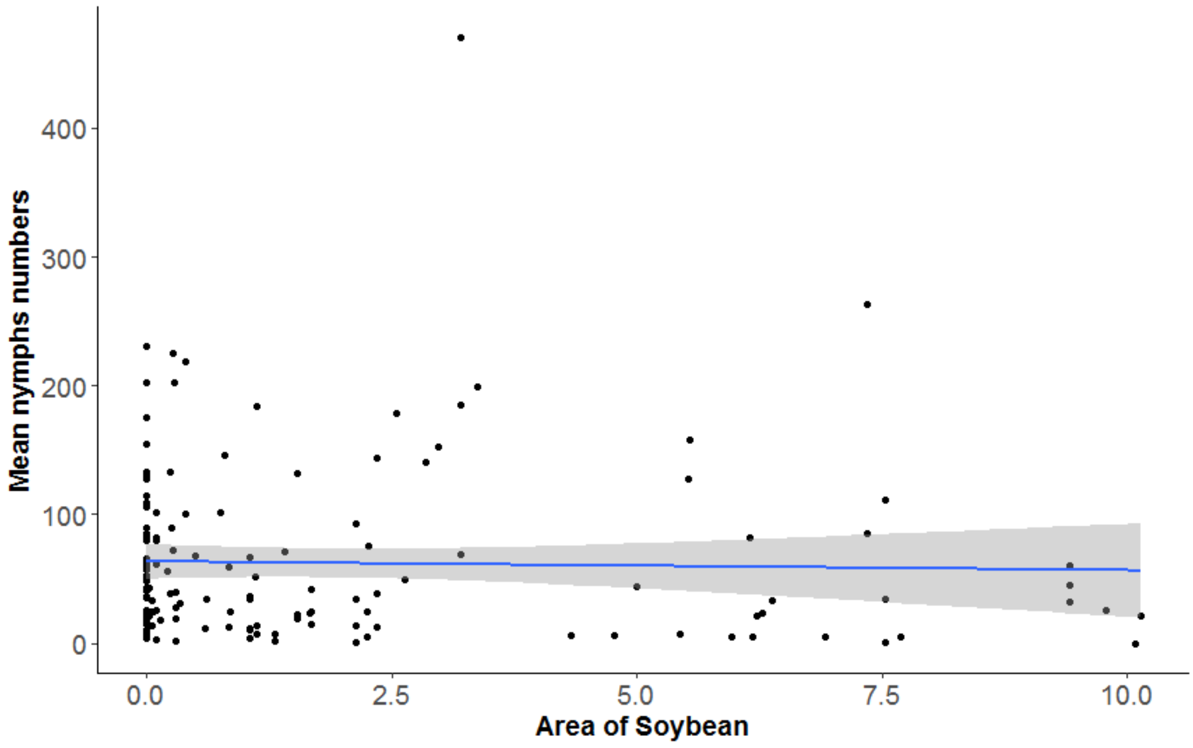

Figure S5. Relationship between area of maize and *Bemisia* nymph abundance in cassava

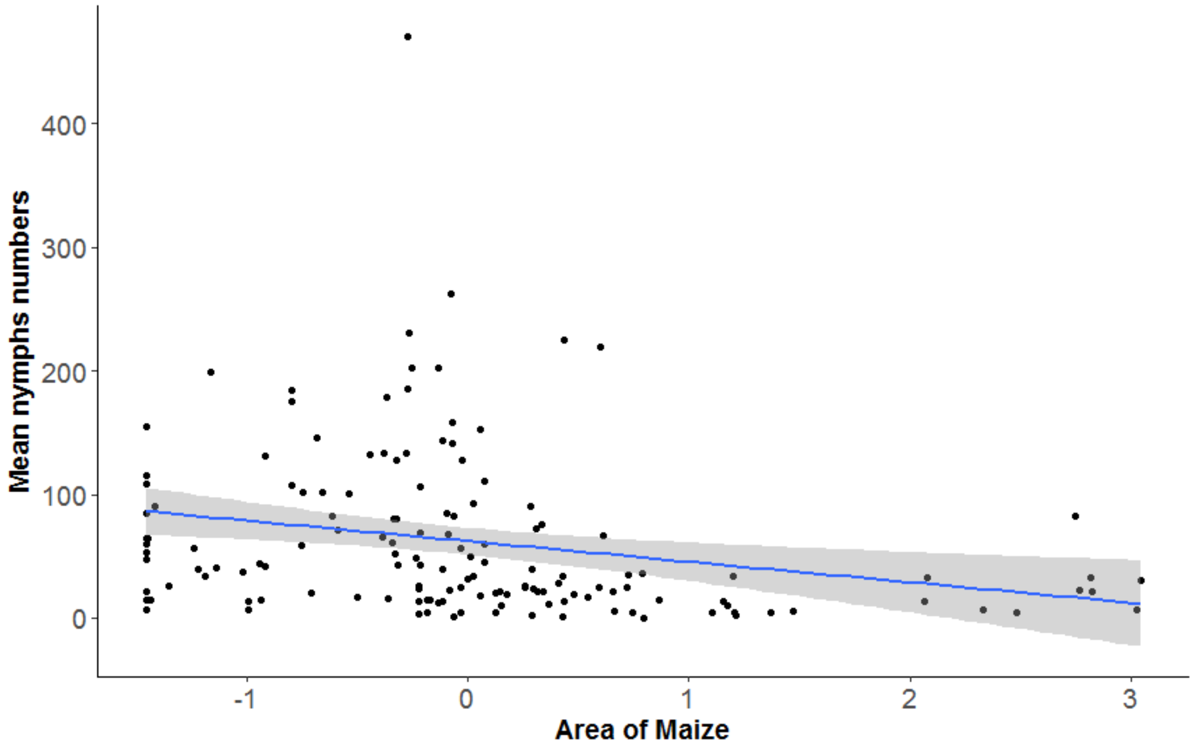

Figure S6. Relationship between area of beans and *Bemisia* nymph abundance in Cassava

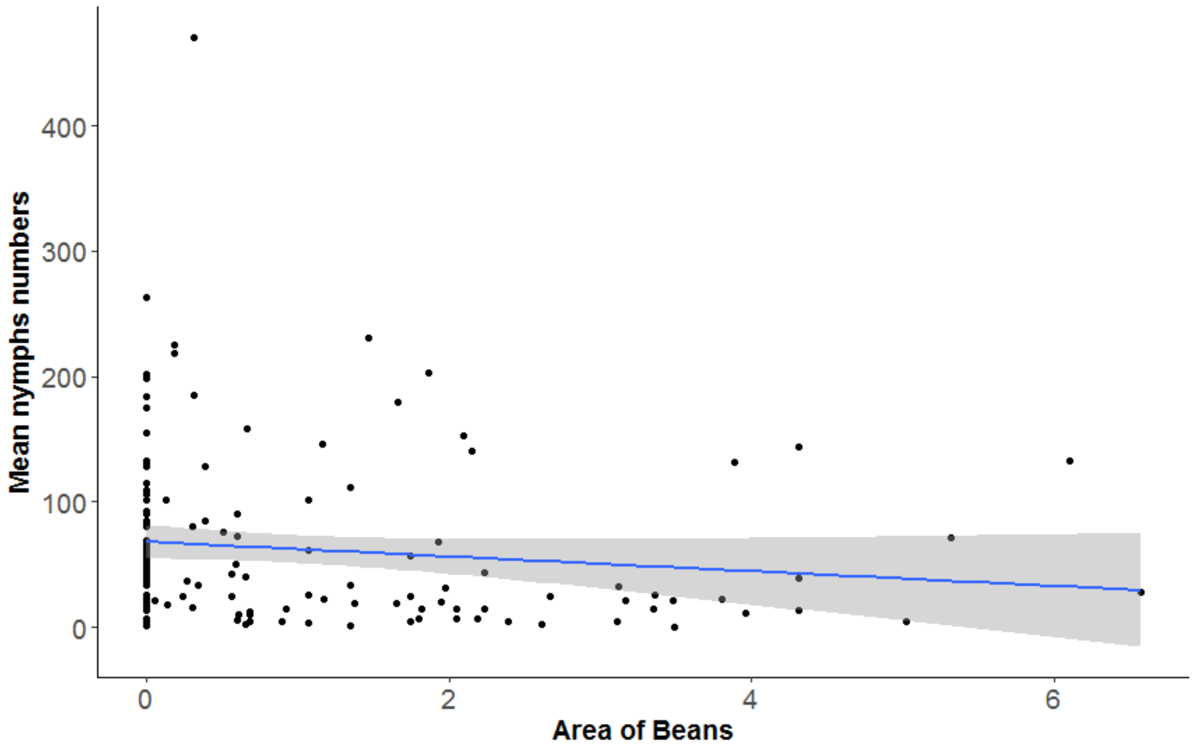

Figure S7. Relationship between area of eggplant and *Bemisia* nymph abundance in cassava

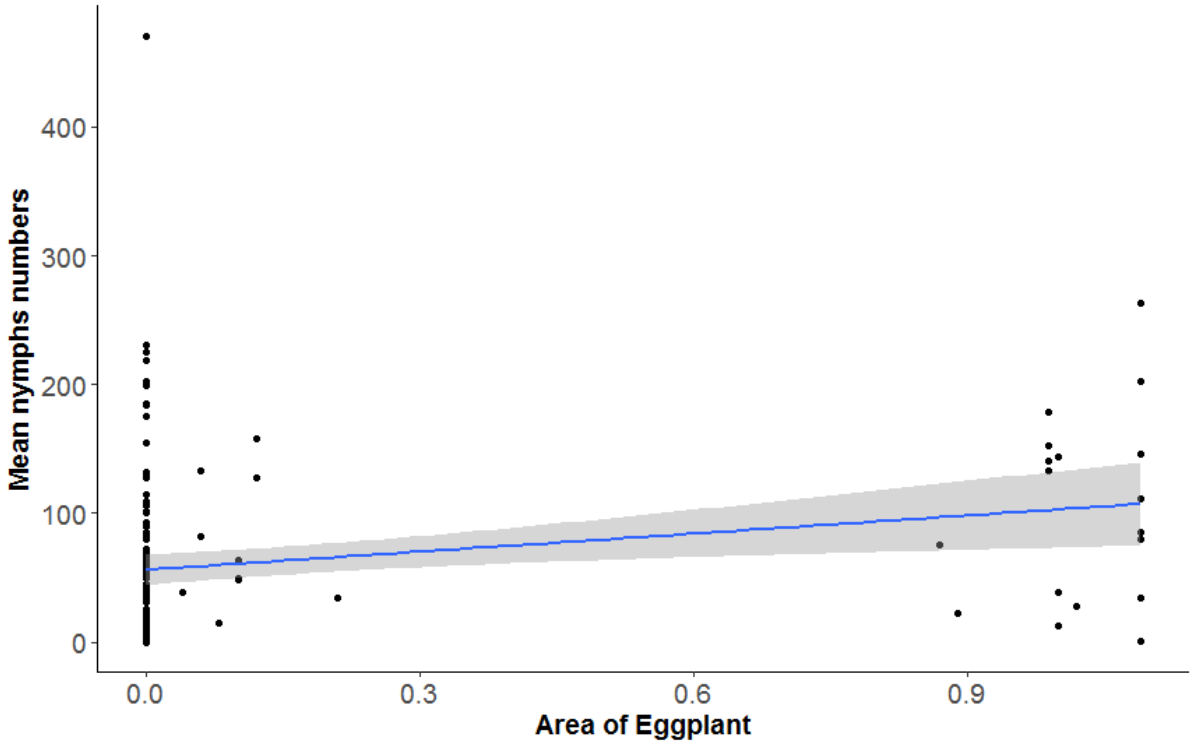

Figure S8. Relationship between area of cocoyam and *Bemisia* nymph abundance in cassava

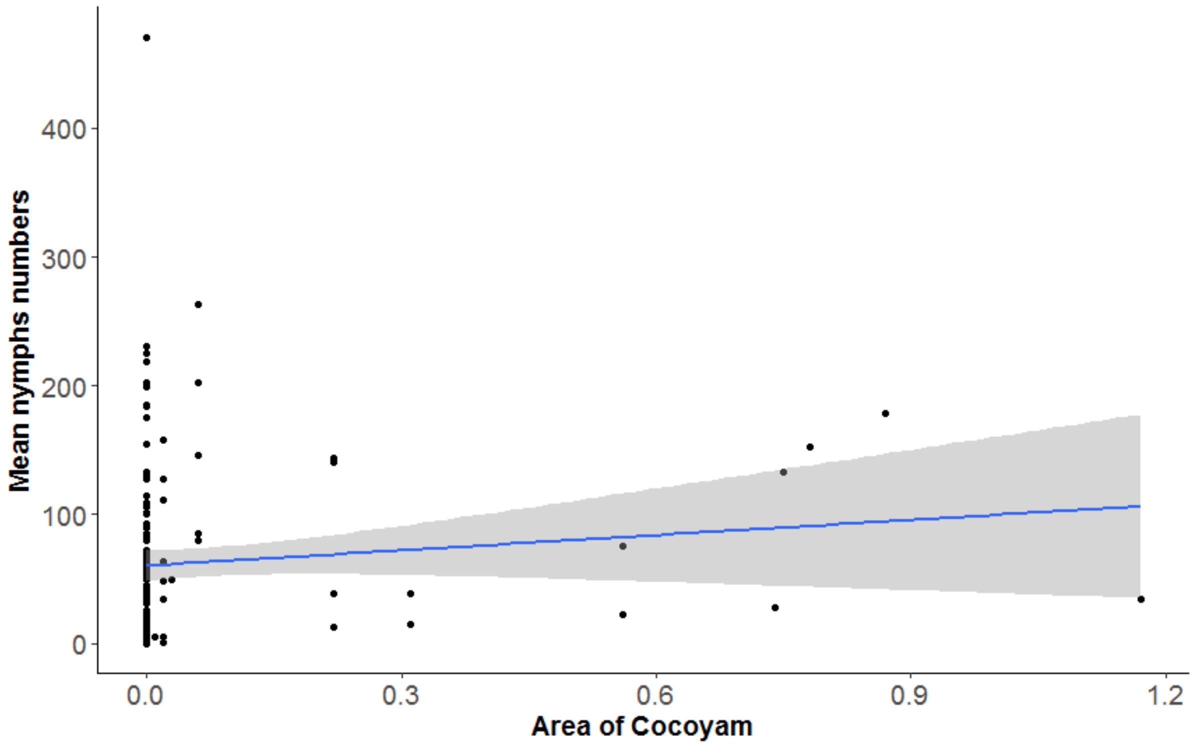

Figure S9. Relationship between area of banana and *Bemisia* nymph abundance in cassava

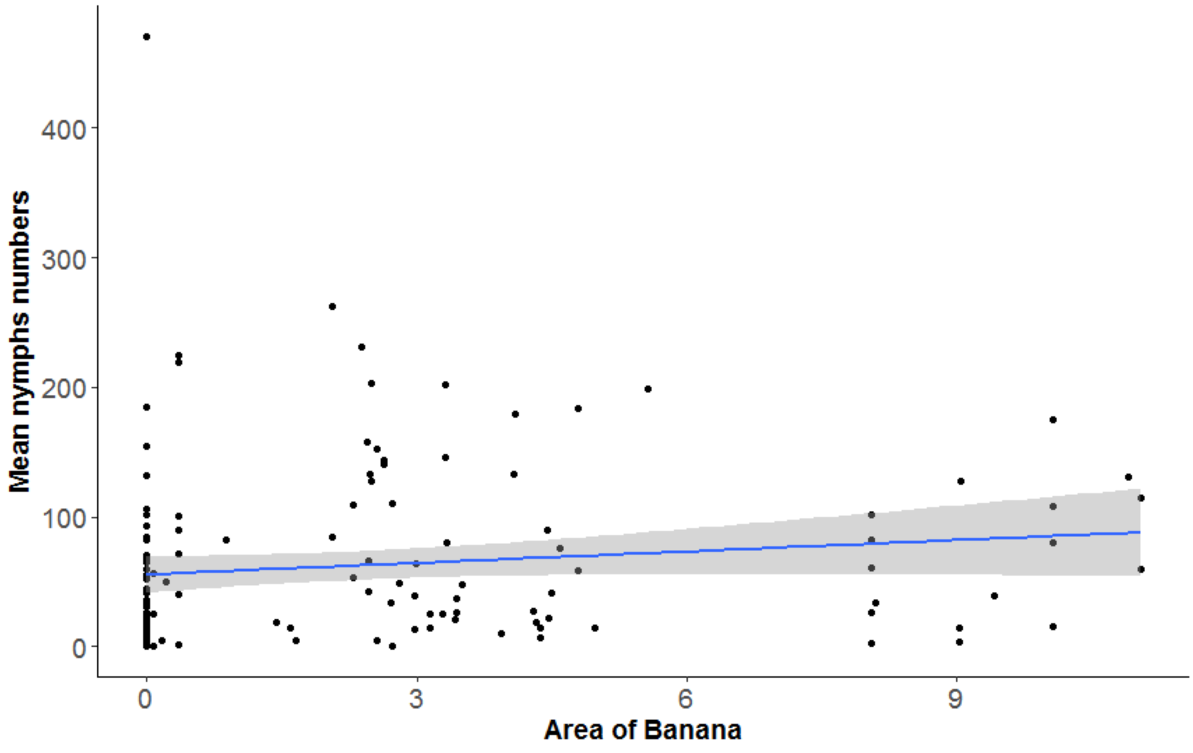

Figure S10. Scatterplot of percent parasitism and area of weeds in the landscape

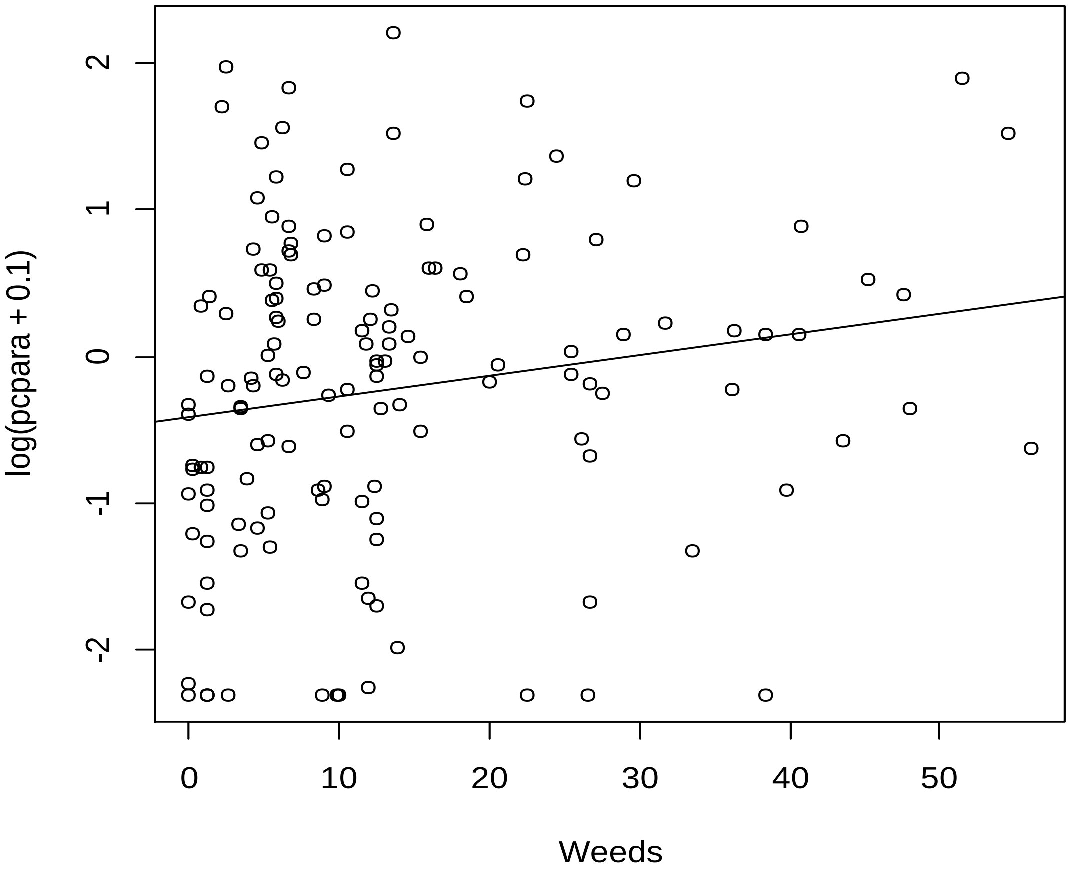

Figure S11. Relationship between area of cassava and non-crop patches in the landscape

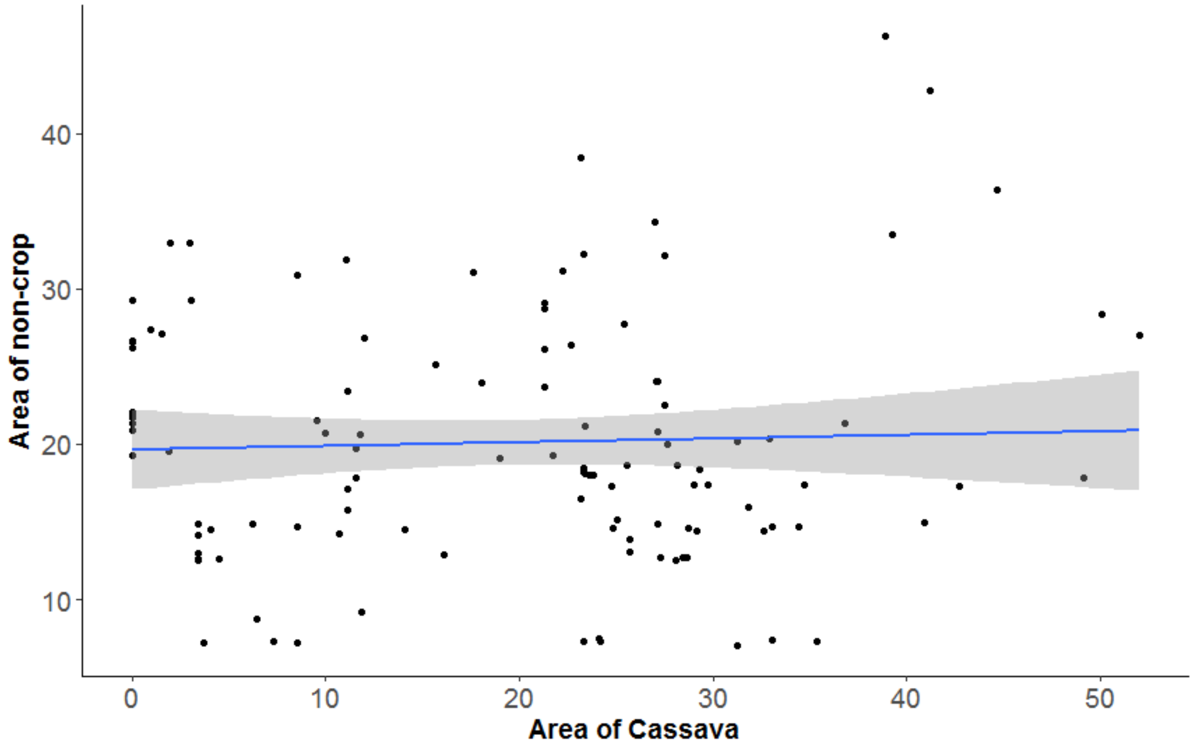

Supplement: Supplementary file 1 [file insects-12-00269-s001.zip › Supplementary Material/Kalyebi-Supplementary Figures-Edited.pdf]
