# Supplementary material for: Within-Season Changes in Land-Use Impact Pest Abundance in Smallholder African Cassava Production Systems
Source: Insects. 2021 Mar 22;12(3):269. doi: 10.3390/insects12030269 (PMC8005198; doi:10.3390/insects12030269)
Supplement: Supplementary file 1 [file insects-12-00269-s001.zip › Supplementary Material/Supplemenraty tables.pdf]

Table S1. Composition of clusters by numbers of landscapes (field-IDs)

Cluster / Field-ID (landscape)	1	2	3	4	5	6	7
1A	12	0	0	0	3	0	0
1B	5	6	0	1	2	1	0
1C	0	0	8	6	0	1	0
1D	9	3	0	0	3	0	0
1E	10	0	2	1	2	0	0
2A	7	2	0	0	4	0	0
2B	3	0	5	4	0	2	0
2C	2	9	1	1	1	0	0
2D	0	0	0	3	0	3	8
2E	4	0	0	1	9	0	0
Totals	52	20	16	17	24	7	8

Table S2. Composition of clusters by months after planting (Map, numbers)

Cluster / MAP	1	2	3	4	5	6	7
1early	16	10	5	1	4	1	2
2peak	16	4	6	1	5	0	3
3mid	12	3	1	7	5	0	2
4late	8	3	4	8	0	6	1
Totals	52	20	16	17	24	7	8
